# Polychlorinated Biphenyl-77 Induces Adipocyte Differentiation and Proinflammatory Adipokines and Promotes Obesity and Atherosclerosis

**DOI:** 10.1289/ehp.10554

**Published:** 2008-03-06

**Authors:** Violeta Arsenescu, Razvan I. Arsenescu, Victoria King, Hollie Swanson, Lisa A. Cassis

**Affiliations:** 1 Graduate Center for Nutritional Sciences; 2 Division of Digestive Diseases and Nutrition; 3 Cardiovascular Research Center; 4 Department of Molecular and Biomedical Pharmacology, University of Kentucky, Lexington, Kentucky, USA

**Keywords:** adipocyte differentiation, aryl hydrocarbon receptor, ectopic lipid deposition, obesity, polychlorinated biphenyl

## Abstract

**Background:**

Obesity, an inflammatory condition linked to cardiovascular disease, is associated with expansion of adipose tissue. Highly prevalent coplanar polychlorinated biphenyls (PCBs) such as 3,3′,4,4′-tetrachlorobiphenyl (PCB-77) accumulate in adipose tissue because of their lipophilicity and increase with obesity. However, the effects of PCBs on adipocytes, obesity, and obesity-associated cardiovascular disease are unknown.

**Objectives:**

In this study we examined *in vitro* and *in vivo* effects of PCB-77 on adipocyte differentiation, proinflammatory adipokines, adipocyte morphology, body weight, serum lipids, and atherosclerosis.

**Methods:**

PCB-77 or 2,2′,4,4,5,5′-hexachlorobiphenyl (PCB-153) was incubated with 3T3-L1 adipocytes either during differentiation or in mature adipocytes. Concentration-dependent effects of PCB-77 were contrasted with those of 2,3,7,8-tetrachlorodibenzo-*p*-dioxin (TCDD). For *in vivo* studies, we treated C57BL/6 wild-type (WT) or aryl hydrocarbon receptor (AhR)^−/−^ mice with vehicle or PCB-77 (49 mg/kg, by intraperitoneal injection) and examined body weight gain. In separate studies, we injected ApoE^−/−^ mice with vehicle or PCB-77 over a 6-week period and examined body weight, adipocyte size, serum lipids, and atherosclerosis.

**Results:**

Low concentrations of PCB-77 or TCDD increased adipocyte differentiation, glycerol–3-phosphate dehydrogenase activity, and expression of peroxisome proliferator–activated receptor γ, whereas higher concentrations inhibited adipocyte differentiation. Effects of PCB-77 were abolished by the AhR antagonist α-naphthoflavone. PCB-77 promoted the expression and release of various proinflammatory cytokines from 3T3-L1 adipocytes. Administration of PCB-77 increased body weight gain in WT but not AhR^−/−^ mice. ApoE^−/−^ mice injected with PCB-77 exhibited greater body weight, adipocyte hypertrophy, serum dyslipidemia, and augmented atherosclerosis.

**Conclusions:**

Our findings suggest that PCB-77 may contribute to the development of obesity and obesity-associated atherosclerosis.

Polychlorinated biphenyls (PCBs), industrial chemicals that were produced and sold in the United States for approximately 50 years (Kimbrough 1985), have continued to bio-accumulate throughout the ecosystem. PCBs are highly lipophilic, with octanol:water partition coefficients of ≥ 10^4^ and, as such, accumulate markedly in lipid-rich tissues (Kodavanti et al. 1998). In lean people, white adipose tissue makes up approximately 15–25% of body weight, and this amount is increased by > 50% in cases of morbid obesity (Mullerova and Kopecky 2006). Thus, the capacity of adipose tissue to accumulate lipophilic PCBs is considerable and would be anticipated to increase with obesity.

The prevalence of obesity has increased at an alarming rate, with 65.4% of the adult population in the United States overweight and 30.5% of adults exhibiting obesity (Flegal et al. 2002; Hedley et al. 2004). Even more alarming, the prevalence of overweight children in the age range of 6–11 years increased from 4.2% in 1963 to 15.3% in 1999–2000 (Ogden et al. 2002). Reaven (1988) noted that several risk factors for cardiovascular disease cluster around an obesity phenotype, termed the metabolic syndrome. Cardiovascular disease has been identified as a primary clinical outcome of patients diagnosed with the metabolic syndrome (Grundy et al. 2004, 2006). Of the cardiovascular diseases associated with obesity, atherosclerosis is a primary cause of death in obese patients (Grundy et al. 2004). Thus, it is important to define mechanisms contributing to the development of obesity and that link obesity to cardiovascular disease.

Interesting recent findings suggest that in nondiabetics with the metabolic syndrome, PCBs were linearly associated with waist circumference (Lee et al. 2007b). Because serum concentrations of PCBs show decreasing trends but obesity is at epidemic proportions, the authors suggested that the toxicity of PCBs may synergistically increase as people become obese (Lee et al. 2007b). Despite the potential for adipocytes to be frequently exposed to PCBs, the specific effects of PCBs on adipocyte function have not been defined. Moreover, the impact of enhanced PCB sequestration in the expanded adipose mass with obesity on the development of obesity-associated cardiovascular diseases is unknown.

Previous results demonstrated that coplanar 3,3′,4,4′-tetrachlorobiphenyl (PCB-77), an aryl hydrocarbon receptor (AhR) ligand, promoted inflammation in endothelial cells (Hennig et al. 1999, 2002a, 2005; Lim et al. 2007). Adipocytes express the AhR (Shimba et al. 1998); however, it is unclear whether coplanar PCBs will also induce expression of inflammatory cytokines in adipocytes. Induction of proinflammatory cytokines by PCBs in adipocytes would be anticipated to promote the development of obesity and obesity-associated cardiovascular disease (Mullerova and Kopecky 2006). In the present study, we contrasted the concentration-dependent effects of coplanar PCB-77 versus 2,3,7,8-tetrachlorodibenzo-*p*-dioxin (TCDD) on adipocyte differentiation using 3T3-L1 adipocytes. Because PCB-77 has been demonstrated to induce inflammatory pathways in endothelial cells (Hennig et al. 1999, 2002b, 2005; Lim et al. 2007), we examined the effects of PCB-77 on proinflammatory and anti-inflammatory adipokine expression in 3T3-L1 adipocytes. To extend these *in vitro* findings, we defined the *in vivo* effects of PCB-77 on body weight gain in C57BL/6 and AhR^−/−^ mice. In separate studies, to determine whether effects of PCB-77 would promote cardiovascular diseases linked to obesity, we defined the effects of PCB-77 on the development of obesity, alterations in serum lipids, and atherosclerosis in apolipoprotein E (apoE)^−/−^ mice.

## Materials and Methods

PCB-77, 2,2′,4,4,5,5′-hexachlorobiphenyl (PCB-153), and TCDD were obtained from AccuStandard Inc. (New Haven, CT). α-Naphthoflavone (α-NF) was obtained from Sigma Aldrich (St. Louis, MO).

### Cell culture and treatment

3T3-L1 mouse embryo fibroblasts, purchased from American Type Culture Collection (Manassas, VA) were maintained in standard Dulbecco’s modified Eagle’s medium (DMEM; Invitrogen, Carlsbad, CA) supplemented with 10% fetal bovine serum (FBS; Gemini Bio-Products, Woodland, CA) and 1% penicillin/streptomycin and subcultured in 60-mm cell culture dishes. Cultured cells (passage number 6 or lower) were allowed to grow to 100% confluence at 37°C in a humidified 5% CO_2_ atmosphere with media changes every 2–3 days. For each experimental parameter measured, assays were performed on duplicate wells of cells within an individual experiment, with a total of three to five experimental replicates.

To define the effects of PCBs on adipocyte differentiation, we incubated preadipocytes with PCB-77 or PCB-153 (3.4 μM) (Hennig et al. 1999, 2002b) 1 day before the induction of differentiation through day 8 of differentiation. Differentiation was induced by incubating cells for 2 days with a cocktail containing insulin (0.1 μM; Sigma, St. Louis, MO), dexa-methasone (1 μM; Sigma), and isobutylmethyl xanthine (0.5 mM, IBMX; Sigma). On the third day, the media was changed to contain only insulin for 1 day, followed by DMEM with 10% FBS for a total of 8 days. Cells were harvested for measurement of oil red O staining, glycerol–3-phosphate dehydrogenase (GPDH) activity, or mRNA quantification of gene expression.

In separate experiments, to contrast concentration-dependent effects of PCB-77 to TCDD, we incubated preadipocytes with vehicle [0.03% dimethyl sulfoxide (DMSO)], PCB-77 (3.4 or 68 μM), or TCDD (0.1, 1, or 10 nM) beginning on day −1 through day 8 of differentiation. Cells were harvested on day 8 for measurement of oil red O staining. To define the role of the AhR in PCB-77− induced regulation of adipocyte differentiation, preadipocytes were incubated with 20 μM α-NF for 30 min before the addition of vehicle, PCB-77 (3.4 μM), or TCDD (0.1 nM) on day 1 through day 8 of differentiation. Cells were harvested on day 8 for measurement of oil red O staining.

To define the effects of PCBs on mature adipocytes, preadipocytes were differentiated using the protocol described above and vehicle, PCB-77 (3.4 μM), or PCB-153 (3.4 μM) was added to the media on day 8 for 24 or 48 hr. Cells were then harvested for mRNA quantification of gene expression and oil red O staining. Cell media was assayed for cytokines as described below.

### Oil red O staining

Cells were washed once with sterile phosphate-buffered saline (PBS) and fixed with 10% formaldehyde in PBS for 5 min at room temperature (Hanlon et al. 2003). Oil red O (0.5% wt/vol stock solution; Sigma) mixed with water (60:40) was used to stain cells (3 mL for 30 min). Dye was extracted from cell culture dishes with isopropanol (1 mL), and the absorbance was measured spectrophotometrically at 510 nm (Ramirez-Zacarias et al. 1992).

### GPDH activity

Cells were rinsed with ice-cold PBS, scraped into 0.2 mL extraction buffer (GPDH assay kit; TAKARA Bio Inc., Shiga, Japan), and centrifuged for 10 min at 40°C. GPDH activity was assayed in the supernatant by monitoring the decrease in absorbance at 340 nm of NADH in the presence of dihydroxyacetone phosphate (Wise and Green 1979).

### RNA isolation and gene expression analysis using real-time polymerase chain reaction (PCR)

Total RNA was extracted from tissues or cells using the phenol guanidine-isothiocyanate method (TRIZOL kit; Invitrogen) per the manufacturer’s instructions. Total RNA (0.4 μg) was reverse transcribed for 1 hr at 55°C with the following components: random decamers, 10× reverse transcription buffer, deoxynucleotide triphosphate mix, ribonuclease inhibitor, and reverse transcriptase (RETROscript; Ambion, Austin, TX). The reverse-transcribed cDNA obtained was then amplified using an iCycler (Bio-Rad, Hercules, CA) with the SYBR Green PCR core reagents kit (Applied Biosystems, Foster City, CA). The ingredients used for the PCR in a total reaction volume of 50 μL included SYBR Green mix (1×), MgCl_2_ (3 mM), dNTP mix (1.25 mM), fluorescein (0.01 μM), primers (0.5 μM), and Amplitaq gold polymerase (2.5 units). Relative quantification of gene expression in the samples was then performed using the standard curve method, and a ratio to 18s rRNA (reference gene) was tabulated. The primers (Qiagen, Valencia, CA) were designed using Primer 3 software (SourceForge, Mountain View, CA), and the sequences are shown in Table 1. The PCR conditions were as follows: 94°C for 5 min, 40 cycles at 94°C for 1 min, 64°C for 1 min, 72°C for 1 min, and a final elongation step of 72°C for 10 min.

### Measurement of PCBs in cells, plasma, and tissue

For the separation of analytes, we used a fully automated Dionex ASE 200 system (Dionex Corporation, Sunnyvale, CA) for assisted solvent extraction and gel permeation chromatography/mass spectrometry (Sipka et al. 2008). This system works by pumping a solvent (hexane) into the top of an electrochemical detection cell, which contains the sample and any in-cell cleanup options. The cell is brought to elevated pressure and temperature, then the extract is forced out of the bottom of the cell and collected in a vial for additional cleanup if necessary. Detection was performed with two microelectron capture detectors; we used Chemstation software (Agilent, Palo Alto, CA) to run the system and interpret the chromatograms. An external standard mixture of PCBs, at known concentrations, was used to test for recovery of the extraction and quantification of PCBs. The limits of detection for PCBs were 0.1 ng/g of tissue (or 0.05 ppm), with coefficient of variability < 3.5% and accuracy (error < 1.5%).

### Measurement of adipokines in media from adipocytes

We measured adipokines in the media of adipocytes using a Luminex multi-analyte detection platform (Mouse Adipocyte Panel; Cayman, Ann Arbor, MI). Values are picograms per milliliter of cell culture media.

### Animal treatments and sample collection

Male C57BL/6 mice or AhR^−/−^ mice (The Jackson Laboratory, Bar Harbor, ME) at 3 months of age (*n* = 10 mice/group) were housed in a pathogen-free environment. Mice were given free access to food and water. All procedures were approved by the Animal Care and Use Committee at the University of Kentucky. Mice were administered vehicle (safflower oil, 0.4 mL) or PCB-77 (49 mg/kg, 0.4 mL) by intraperitoneal (ip) injection for a total of four injections (two in week 1, two in week 4) during the 6-week study duration. Body weight was measured weekly. In separate studies, 3-month-old male ApoE^−/−^ mice bred in-house to C57BL/6 mice from stock originally purchased from The Jackson Laboratory (*n* = 10 mice/group) were administered vehicle (safflower oil, 0.4 mL ip) or PCB-77 (49 mg/kg, 0.4 mL ip) using the same experimental protocol (a total of four injections: two in week 1, two in week 4) during the 6-week study duration. At the study end point, mice were anesthetized with ketamine/xylazine (100/10 mg/kg ip) for blood and tissue harvest.

### Serum cholesterol measurement

We determined serum cholesterol concentrations and lipoprotein cholesterol distributions in ApoE^−/−^ mice in each treatment group as described previously (Daugherty et al. 2000; Manning et al. 2003).

### Histology

A portion of each tissue (mesenteric fat, liver) from ApoE^−/−^ mice in each treatment group was fixed in 4% para-formaldehyde overnight, embedded in paraffin, and cut in serial sections (5 μm). Sections were deparaffinized and stained with hematoxylin and eosin. Adipocyte area was quantified in three sections for each mouse (three fields per section) using an Olympus BX51 microscope and Olympus U-CMAD3 digital camera (Olympus America, Center Valley, PA) and Image-Pro Plus 4.5 software (Media Cybernetics Inc., Bethesda, MD).

### Quantification of atherosclerosis

Frozen aortic root tissues from ApoE^−/−^ mice in each treatment group were sectioned and discarded until the aortic sinus was reached. Tissue samples (10 μM) were subsequently sectioned and placed on slides (Probe-on Plus; Fisher Scientific, Pittsburgh, PA). From the start of section acquisition at the aortic sinus, each section was retained and sequentially placed on 8 slides. A total of 10 slides, each having approximately nine sections 90 μm apart covering approximately 720 μm of the root, were stained for lipid using oil red O. Using a Digital DXM camera (Nikon Instruments Inc., Melville, NY) mounted on a microscope, images (40×) were taken and the area of lesions quantified using Image-Pro Software Plus 4.5.

### Statistical analysis

Data are expressed as mean + SE. Data were tested for normality and equal variance. For *in vitro* studies comparing effects of PCB-77 with PCB-153, data were analyzed using one-way analysis of variance (ANOVA) (GraphPad Prism, version 4; GraphPad Software Inc., San Diego, CA). For studies examining concentration dependence of PCB-77 or TCDD, data were analyzed by two-way ANOVA, with toxic compound and concentration as between-group factors. For post hoc analysis, data were analyzed using Tukey’s test, with significance at *p* < 0.05. For studies in C57BL/6 and AhR^−/−^ mice, body weight gain was analyzed by one-way ANOVA with repeated measures on time. For studies in ApoE^−/−^ mice, with the exception of body weight, data were analyzed by Student’s independent *t*-test. Significance was accepted at *p* < 0.05.

## Results

### PCB-77 promotes 3T3-L1 adipocyte differentiation and the expression of proinflammatory adipokines

**Figure 1:**
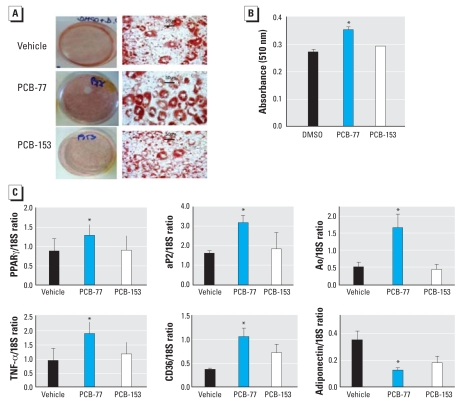
Effect of PCB-77 and PCB-153 on adipocyte differentiation and mRNA expression of proinflammatory adipokines. (*A*) Macroscopic (left) and microscopic (right; 40× magnification) images of oil red O–stained adipocytes. (*B*) Quantification of oil red O staining. (*C*) mRNA expression of PPARγ, aP2, Ao, TNF-α, CD36, and adiponectin. Data are mean ± SE from five individual experiments. *Significantly different from vehicle (*p* < 0.05).

We examined the effects of coplanar PCB-77 and nonplanar PCB-153 at equivalent concentrations (3.4 μM) (Hennig et al. 1999, 2002b) on the differentiation of 3T3-L1 adipocytes. PCB-77, but not PCB-153, increased oil red O staining (Figure 1A). Expression (mRNA) of peroxisome proliferator–activated receptor γ (PPARγ), a master regulator of adipocyte differentiation, and its downstream target, adipocyte fatty acid–binding protein (aP2), were increased by PCB-77, but not by PCB-153 (Figure 1C). PCB-77 resulted in an increase in mRNA expression of angiotensinogen (Ao), tumor necrosis factor α (TNF-α), and differentiation-36 (CD36) (Figure 1C). In contrast, adiponectin mRNA expression and concentrations in cell media (Table 2) were decreased by PCB-77. In addition, PCB-77 increased the concentrations of monocyte chemoattractant protein-1 (MCP-1) and keratinocyte chemoattractant-1 (KC-1) in cell media (Table 2).

**Figure 2:**
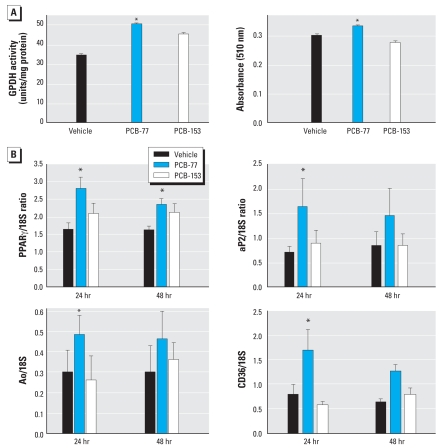
Effect of PCB-77 and PCB-153 on mRNA expression of proinflammatory adipokines in mature 3T3-L1 adipocytes. (*A*) GPDH activity (left) or oil red O staining (right). (*B*) mRNA expression of PPARγ, aP2, Ao, or CD36. Data are mean ± SE from five individual experiments. *Significantly different from vehicle (*p* < 0.05).

The ability of PCB-77 to promote adipokine expression when incubated with preadipocytes could result from enhanced adipocyte differentiation. We therefore examined the effect of PCB-77 and PCB-153 on mRNA abundance of adipokines when incubated with mature differentiated adipocytes (day 8) for 24 or 48 hr. The late-stage differentiation marker, GPDH activity, was increased by PCB-77 (Figure 2A; 48-hr incubation). In addition, oil red O staining was increased by PCB-77 (Figure 2A; 48-hr incubation). After 24 hr incubation, PCB-77 increased the mRNA expression of PPARγ, Ao, aP2, and CD36 (Figure 2B).

### The concentration-dependent effects of PCB-77 versus TCDD and the role of the AhR

**Figure 3:**
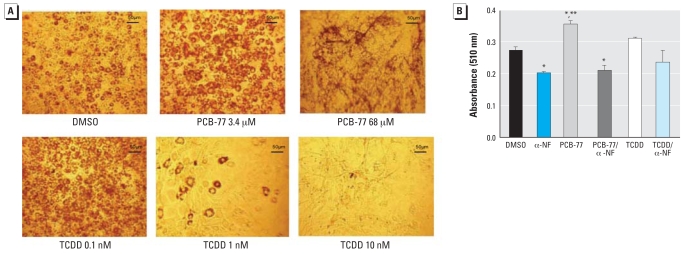
Effects of low and high concentrations of PCB-77 and TCDD on adipocyte differentiation (effects of PCB-77 are AhR mediated). (*A*) Oil red O staining (representative images from three individual experiments; 20× magnification). (*B*) Quantification of oil red O staining. Data are mean ± SE from three experiments. *Significantly different from vehicle (*p* < 0.05). **Significantly different from PCB-77/α-NF (*p* < 0.05).

PCB-77 has affinity for AhRs; TCDD, a potent AhR ligand, has been shown to inhibit adipocyte differentiation at concentrations > 1 nM (Alexander et al. 1998). We therefore compared the concentration-dependent effects of PCB-77 (3.4, 34, and 68 μM) with TCDD (0.1, 1, and 10 nM) on the differentiation of 3T3-L1 adipocytes. We chose PCB-77 concentrations based on a toxic equivalency factor (TEF; compares the relative potency of a compound with TCDD, assigned as 1.0 TEF) of 0.0001 (Van den Berg et al. 2006). Thus, at a concentration of 3.4 μM, PCB-77 would be anticipated to be approximately equipotent to 0.1 nM TCDD. At low concentrations of PCB-77 (3.4 μM), oil red O staining increased (Figure 3). Modest elevations in oil red O staining with low concentrations of TCDD (0.1 nM) were not significant compared with vehicle (Figure 3B). Higher concentrations of each toxic compound resulted in a reduction in oil red O staining (Figure 3A). Because adipocytes were not clearly visible with high concentrations of TCDD or PCB-77, we did not quantify oil red O staining at high concentrations. The AhR antagonist α-NF decreased oil red O staining (Figure 3B). In addition, α-NF decreased PCB-77 (3.4 μM)-induced elevations in oil red O staining. In contrast to findings with lower concentrations of PCB-77 (3.4 μM), a higher concentration of PCB-77 (68 μM) and TCDD resulted in a reduction in release of proinflammatory adipokines from adipocytes when incubated with preadipocytes during differentiation (Table 2).

### In vivo administration of PCB-77 increases body weight gain in C57BL/6, but not in AhR^−/−^ mice

**Figure 4:**
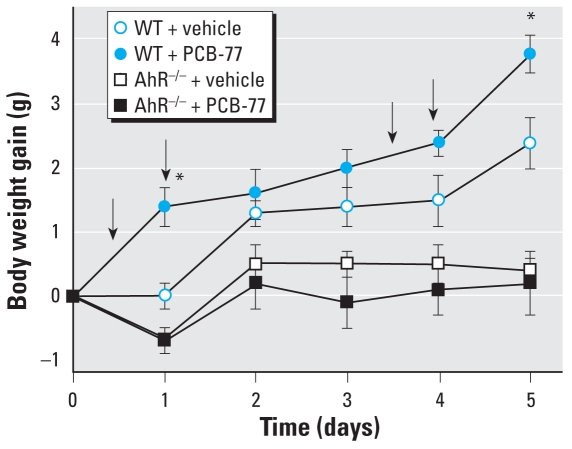
Effect of PCB-77 on body weight gain in WT C57BL/6 and AhR^−/−^ mice. Arrows indicate times of vehicle or PCB-77 injections. Data are mean ± SE from 10 mice per group. *Significantly different from vehicle, within genotype.

To determine if the *in vitro* effects of PCB-77 occur with *in vivo* exposure and whether these effects are AhR-mediated, we administered PCB-77 to C57BL/6 wild-type (WT) and AhR^−/−^ mice. AhR-deficient mice administered vehicle exhibited lower body weight gain than vehicle-injected WT controls (Figure 4). In WT mice administered PCB-77, body weight gain increased at 1 and 5 weeks compared with vehicle. In contrast, body weight gain did not increase in AhR^−/−^ mice administered PCB-77.

### In vivo administration of PCB-77 results in increased body weight and adipose mass, elevated serum cholesterol concentrations, and increased atherosclerosis in ApoE^−/−^ mice

**Figure 5:**
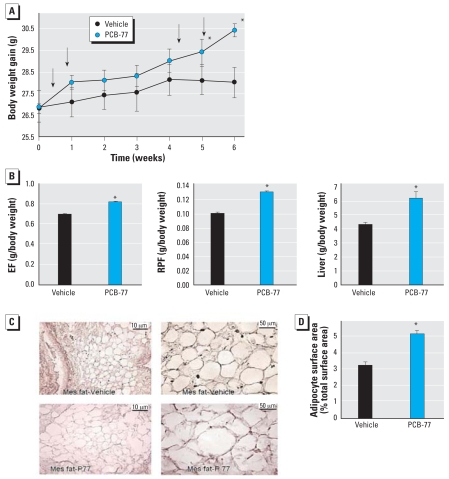
Effect of PCB-77 on body weight, adipose mass, and liver weight in ApoE^−/−^ mice. (*A*) Body weight (arrows indicate times of vehicle or PCB-77 injections). (*B*) Weights of EF, RPF, and liver (percentage of body weight). (*C*) Tissue sections from mesenteric adipose tissue. (*D*) Quantification of adipocyte surface area (percentage of total section surface area; right) in representative tissue sections of mesenteric (Mes) adipose tissue. Data are mean ± SE from 10 mice per group. *Significantly different from vehicle (*p* < 0.05).

To determine whether elevations in body weight would promote obesity-associated atherosclerosis, we defined effects of PCB-77 in hyper-cholesterolemic ApoE^−/−^ mice. Body weight (28.0 ± 0.70 g for vehicle; 30.4 ± 0.30 g for PCB-77; *p* < 0.05; Figure 5A) and weight gain (1.28 ± 0.4 g for vehicle; 2.62 ± 0.30 g for PCB-77; *p* < 0.05) were higher in PCB-77− treated mice compared with vehicle-treated mice. Increases in body weight were associated with increases in the mass of gonadal [epididymal fat (EF)] and visceral [retroperitoneal fat (RPF)] adipose tissue as well as the liver (Figure 5B). In tissue sections from mesenteric white adipose tissue, adipocytes from mice injected with PCB-77 were hypertrophied compared with those from vehicle-treated mice (Figure 5C,D). The concentration of PCB-77 in EF was higher in mice administered PCB-77 (55 ± 8 μg/g) than in vehicle-treated animals (< 0.6 μg/g). Moreover, PCB-77 was not detected in the plasma of vehicle-injected mice, but it was within detectable concentrations in mice administered PCB-77 (0.365 μg/g).

**Figure 6:**
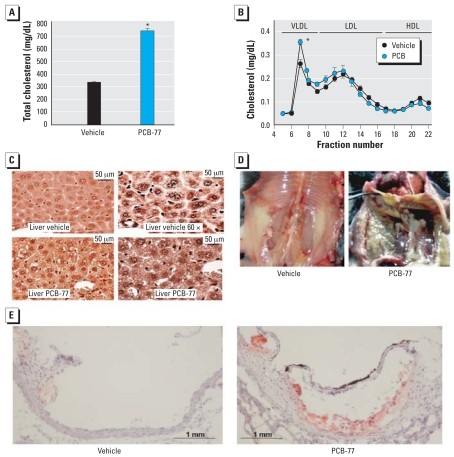
Effects of PCB-77 on total serum cholesterol and VLDL cholesterol concentrations, lipid deposition within the liver and abdominal cavity, and atherosclerosis in ApoE^−/−^ mice. (*A*) Total serum cholesterol concentrations (*n* = 10 mice per group). (*B*) Lipoprotein cholesterol distributions (*n* = 4 mice per group). (*C*) Representative tissue sections from livers of mice injected with vehicle or PCB-77. (*D*) Abdominal cavity from mice administered vehicle or PCB-77. (*E*) Aortic root sections stained with oil red O from vehicle or PCB-77− injected mice. *Significantly different from vehicle (*p* < 0.05).

Total serum cholesterol concentrations were markedly increased in ApoE^−/−^ mice injected with PCB-77 compared with vehicle (Figure 6A). Elevations in serum cholesterol concentrations in PCB-77− treated mice were predominantly in very low-density lipoprotein (VLDL) cholesterol (Figure 6B). Moreover, compared with vehicle-treated mice, tissue sections of liver from mice administered PCB-77 exhibited lipid-laden vacuoles (Figure 6C). Unexpectedly, administration of PCB-77 resulted in marked deposition of lipid within the abdominal cavity (Figure 6D). The extent of atherosclerosis was low in aortic root sections of ApoE^−/−^ mice administered vehicle (Figure 6E). Administration of PCB-77 to ApoE^−/−^ mice increased atherosclerosis in aortic root sections (0.002 μm^2^ mean lesion area in PCB-77− injected mice; nondetectable in vehicle controls; *p* = 0.032; Figure 6E).

## Discussion

It has long been recognized that the high lipophilicity of PCBs and related toxic compounds favors their localization to adipose tissue. However, their effects on adipocyte function have not been established. Our results suggest that lower concentrations of coplanar PCBs, acting as ligands of the AhR, promote adipocyte differentiation and increase the expression of proinflammatory adipokines. In contrast, higher concentrations, similar to TCDD, inhibit adipocyte differentiation. Importantly, when administered to WT, but not AhR-deficient mice, PCB-77 caused an increase in body weight gain. These results confirm *in vitro* findings and suggest that effects of PCB-77 are AhR mediated. In hypercholes-terolemic ApoE^−/−^ mice, PCB-77 increased body weight associated with adipocyte hypertrophy, expanded adipose mass, and increased serum cholesterol concentrations and ectopic lipid deposition. These effects of PCB-77 were associated with increased atherosclerosis. These results suggest that exposure to PCB-77, a dioxin-like PCB, at relatively low levels may promote the development of obesity and obesity-associated atherosclerosis.

PCBs are highly lipophilic, with octanol:water partition coefficients of ≥ 10^4^, making their accumulation in nonlipid material negligible. In studies aimed at determining the congener-specific distribution of PCBs with chronic exposure, adult rats were treated five times per week for 4 weeks by gavage with Aroclor 1254 in corn oil (Kodavanti et al. 1998). Total PCB (parts per milllion) accumulation in fat was 551 μg/g; the second-highest tissue accumulation of PCBs was in liver (38 μg/g). The mean blood:liver:fat tissue ratios were 1:22:359, similar to previously observed results for PCB-153 (Wyss et al. 1986) or 2,2′3′,4,4′,5,5′-heptachlorobiphenyl (PCB-180) (Koss et al. 1993). In the present study, adipocyte differentiation and proinflammatory adipokine expression were induced in 3T3-L1 adipocytes by PCB-77 but not by PCB-153. These PCB congeners differ slightly in their oil:water partition coefficients, favoring greater lipophilicity of PCB-153; however, both PCBs would be anticipated to accumulate in adipose tissue. A lack of effect of PCB-153 may have resulted from greater sequestration of this more-lipophilic PCB in the triacylglycerol droplet of the adipocyte, leaving less PCB-153 available to act at adipocyte target proteins. However, given that PCB-77 and TCDD exhibited similar effects and that TCDD has a high octanol:water partition coefficient comparable to PCB-153 (6.7 vs. 6.8, respectively), other mechanisms most likely mediated differences in effects of PCB-77 and PCB-153. Specifically, results from this study demonstrate that interactions with the AhR, for which both PCB-77 and TCDD possess affinity (but PCB-153 has low affinity), contributed to differences in the effects of these PCBs.

Phillips et al. (1995) demonstrated that treatment of 3T3-L1 preadipocytes with 10 nM TCDD during the first 2 days of induction of differentiation resulted in a reduction in the number of fat cell colonies. The effect of TCDD to inhibit adipocyte differentiation was blocked by treatment with an AhR antagonist. To extend these findings to the *in vivo* situation, Brodie et al. (1996) demonstrated that a single high dose (175 μg/kg) of TCDD administered to rats resulted in inhibition of adipocyte differentiation. Additional studies by this group demonstrated that high-dose TCDD treatment in rats resulted in a reduction in preadipocyte differentiation to mature adipocytes, which was associated with decreases in transcription factor mRNAs (PPARγ, aP2, C/EBPβ) normally elevated during adipocyte differentiation (Brodie et al. 1997). Using the 3T3-L1 adipocyte differentiation system, Shimba et al. (1998) demonstrated that the level of AhR protein decreased with ongoing adipocyte differentiation. Using mouse embryo fibroblasts, Alexander et al. (1998) demonstrated that treatment with 10 nM TCDD inhibited differentiation in cells from WT but not AhR^−/−^ mice. At high concentrations (approximately 10-fold greater than the dissociation constant), the literature and data from this study demonstrate that TCDD decreases adipocyte differentiation, commensurate with the wasting syndrome demonstrated from high-dose toxicity.

Several epidemiologic studies have suggested a link between exposure to dioxin, or PCBs, and diabetes (Bertazzi et al. 1997; Calvert et al. 1999; Codru et al. 2007; Cranmer et al. 2000; Lee et al. 2006, 2007a; Vasiliu et al. 2006). Most of these studies represent findings from type 2 diabetics with obesity; however, increased relative risk for exposure to toxic compounds and the development of diabetes remains when data were adjusted for differences in body mass index across study populations. Interestingly, the association between persistent organic pollutants and diabetes was much stronger in obese subjects compared with lean subjects (Lee et al. 2006). Moreover, in nondiabetic adults, results from the National Health and Nutrition Examination Survey (1999–2002) (Lee et al. 2007b) demonstrated a linear positive relationship between serum concentrations of PCBs, including dioxin-like PCBs, and waist circumference. Unfortunately, similar epidemiologic studies have not been performed to define whether exposure to PCBs increases the risk for development of obesity or obesity-associated cardiovascular disease, including atherosclerosis.

Obesity, a condition that would predictably increase the body burden of lipophilic PCBs, is associated with an elevation in the systemic concentrations of a variety of factors that are produced and released from adipocytes (Lago et al. 2007; Lau et al. 2005). Many of these factors have been linked to diseases clustering around an obesity phenotype, including coronary artery disease, the primary cause of death in the obese population. Thus, factors that regulate adipokine secretion from adipocytes may influence not only obesity but also obesity-associated atherosclerosis. The present results demonstrate that PCB-77 promotes the expression and secretion of a variety of proinflammatory adipokines from 3T3-L1 adipocytes and decreases the expression of adiponectin, an anti-inflammatory adipokine. Although previous studies have demonstrated proinflammatory effects of PCBs in various cell types (Choi et al. 2003; Eum et al. 2006; Hennig et al. 1999, 2002b; Ramadass et al. 2003), to our knowledge, this is the first report demonstrating that PCBs can promote the production and elaboration of these factors from adipocytes. Interestingly, Vogel et al. (2007) recently reported that a single injection of TCDD to C57BL/6 mice resulted in an increase in MCP-1 and KC-1 in liver and adipose tissue. However, enhanced expression of these adipokines in adipose tissue was associated with increased expression of the macrophage marker F4/80, suggesting that this effect may have resulted from enhanced macrophage infiltration into adipose tissue. Our results extend these findings by demonstrating that PCB-77 can act directly on 3T3-L1 adipocytes to promote the mRNA abundance and secretion of several pro-inflammatory adipokines.

To extend results from *in vitro* experiments to an *in vivo* model, we administered PCB-77 to WT or AhR-deficient mice or to hyper-cholesterolemic ApoE^−/−^ mice. In previous studies, C57BL/6 mice that received 30 mg/kg/day of PCB-77 consumed in food for a total of 16 weeks exhibited a reduction in body weight (Goodwill et al. 2007). Thus, chronic dosing with high doses of PCB-77 appears to mimic the wasting syndrome that results from toxic TCDD exposure. In the present study, we injected mice four times (49 mg/kg per dose) with PCB-77 over a 6-week period; although this was a higher daily dose than used by Goodwill et al. (2007), it was far less cumulative. Our choice of PCB-77 dose was based on previous studies demonstrating that this dose exhibits proinflammatory effects on endothelial cells when injected into mice (Hennig et al. 2002b) and is classified as a moderate exposure dose in experimental animals (Brouwer et al. 1999; Jensen et al. 2001; Wassermann and Wassermann 1979). In agreement, PCB-77 levels in adipose tissue of mice from this study were far lower than those reported in adipose tissue from rats administered Arochlor [55 vs. 551 μg/g; (Kodavanti et al. 1998)]. Our results demonstrate that in contrast to high-dose PCB-77 dosing *in vivo*, lower doses exhibit an opposite effect to increase body weight. Moreover, these effects were AhR mediated. The ability of PCB-77 to increase adipose mass with associated adipocyte hypertrophy may relate to the *in vitro* effects of PCB-77 to promote adipocyte differentiation. Alternatively, the ability of PCB-77 to increase CD36 mRNA abundance in adipocytes may have promoted lipid uptake and contributed to adipocyte hypertrophy.

Previous investigators demonstrated that dietary exposure to PCBs results in fatty liver and hypercholesterolemia in rats (Kato et al. 1980; Nagaoka et al. 1990; Quazi et al. 1983). These effects were primarily attributed to an increase in hepatic cholesterol synthesis. In the present study, a low dose of PCB-77 resulted in a marked increase in serum cholesterol concentrations in ApoE^−/−^ mice, with predominant increases in VLDL cholesterol. These results extend previous findings by demonstrating that in a mouse model exhibiting hyper-cholesterolemia and atherosclerosis, marked elevations in serum cholesterol concentrations are induced by PCB-77. Moreover, elevations in VLDL cholesterol by PCB-77 in this study were associated with increased atherosclerosis. To our knowledge, this is the first study that has directly examined the effects of PCB exposure on experimental atherosclerosis.

In conclusion, at low exposure levels, coplanar PCB-77 promoted adipocyte differentiation and proinflammatory adipokine expression. In contrast, both PCB-77 and TCDD inhibited adipocyte differentiation at higher concentrations. Effects of PCB-77 to promote adipocyte differentiation and regulate body weight were AhR mediated. Importantly, when administered *in vivo* to ApoE^−/−^ mice at a moderate dose, PCB-77 resulted in an increase in body weight, adipose mass and adipocyte area, serum cholesterol concentrations, and atherosclerosis. These results suggest that low-level exposure to coplanar PCBs may contribute to the development of obesity and to obesity-associated atherosclerosis.

## Figures and Tables

**Table 1:** Primer sequences for real-time PCR.

Gene	Forward	Reverse
18S	CTCTGTTCCGCCTAGTCCTG	AATGAGCCATTCGCAGTTTC
PPARγ	GATGGAAGACCACTCGCATT	AACCATTGGGTCAGCTGCTCTTG
aP2	TCACCTGGAAGACAGCTCCT	AAGCCCACTCCCACTTCTTT
CD36	AGGTCCTTACACATACAGAGTTCG	GGACTTGCATGTAGGAAATGTGGA
Adiponectin	GTTGCAAGCTCTCCTGTTCC	ATCCAACCTGCACAAGTTCT
Angiotensinogen	TCTCTTACCCCTGCCCTCT	GAACCTCTCATCGTTCCTTG

Abbreviations: aP2, adipocyte fatty acid–binding protein; PPARγ, peroxisome proliferator–activated receptor γ.

**Table 2:** Adipokines released from 3T3-L1 adipocytes incubated with vehicle or PCB-77.

	MCP-1 (pg/mL)	Adiponectin (pg/mL)	Leptin (pg/mL)	IL-6 (pg/mL)	KC-1 (pg/mL)
Vehicle	3,069 ± 297	43,200 ± 1,400	7.78 ± 2.3	5.6 ± 0.1	2,912 ± 321
PCB-77 (3.4 μM)	7,217 ± 1,238*	16,550 ± 250*	4.98 ± 1.7	8.6 ± 2.3	4,820 ± 794*
PCB-77 (68 μM)	1,510 ± 128	129 ± 80*	< 3.00	2.8 ± 0.1	< 3.00
TCDD (10 nM)	2,810 ± 430	1,106 ± 325*	< 3.00	3.8 ± 0.7	< 3.00

IL-6, interleukin 6. Data are mean ± SE from three per treatment.

* Significantly different from vehicle (*p* < 0.05).
